# Fibrous hamartoma of infancy with sarcomatous transformation: an unusual morphology

**DOI:** 10.4322/acr.2021.380

**Published:** 2022-04-28

**Authors:** Gargi Kapatia, Debajyoti Chatterjee, Kirti Gupta, Amita Trehan

**Affiliations:** 1 Post Graduate Institute of Medical Education & Research, Department of Histopathology, Chandigarh, India; 2 Post Graduate Institute of Medical Education & Research, Department of Pediatrics, Chandigarh, India

**Keywords:** Hamartoma, Neoplasms, Fibrous Tissue, Neoplasms, Connective Tissue, Neoplasms, Connective and Soft Tissue

## Abstract

**Background:**

Fibrous hamartoma of infancy (FHI) is a rare soft tissue lesion arising as a subcutaneous mass involving the axilla, trunk, and upper arm in infants and children <2yrs. Sarcomatous transformation in FHI is described in anecdotal cases in the literature.

**Case Report:**

We describe one such example arising as a mass in the lower back in a 3-month-old infant. On histology, the tumor contained classic triphasic morphology; however, brisk mitotic activity noted at multiple foci was diagnostically challenging to categorize. The tumor was evaluated for *ETV6-NTRK3* fusion to exclude other common differentials.

**Conclusion:**

While FHI may be frequently encountered in infants, rare sarcomatous transformation are known to occur and merits special attention as it can be misdiagnosed. Also, a close follow-up is warranted as the lesion is known to recur locally.

## INTRODUCTION

Fibrous hamartoma of infancy (FHI) is a benign soft tissue lesion recognized by its triphasic morphology since the early days.^1^ It is frequently known to have its onset during the first two years of life; subsequently, several reports indicated deviation from its classic demographics with expanded age range and anatomic distribution outside its classic locations.^2^^-^4 While the natural course is classically benign with an excellent prognosis, the histogenesis of this tumor is still elusive, with the lesion being described as neoplastic, to being hamartomatous or a malformation.^5^ Nevertheless, the favorable prognosis is often marred by local recurrences with rare sarcomatous transformation on record.^6^ We describe one such example of FHI as a congenital mass in a three-month-old infant with distinctive morphology albeit with sarcomatous transformation and discuss the differentials. Such lesions should be correctly diagnosed as local recurrences are known to occur.

## CASE REPORT

A 3-month-old boy presented with a mass in the lower back, which was present since birth. It initially measured 3x2 cm with a tuft of hair and gradually increased in size. There was no associated bowel or bladder disturbance, no paucity in lower limb movements, or any discharge history from the swelling. On examination, the mass measured 10x15 cm and was soft in consistency, non-tender, non-fluctuant. It extended from L1 to the right gluteal region. Anthropometric parameters were normal for his age. Investigations revealed normal hemogram and normal biochemical parameters, including alpha-fetoprotein levels (85.9 IU/ml; reference range 5-877 IU/ml). The magnetic resonance imaging (MRI) of the lumbosacral spine revealed large subcutaneous mass in the lumbosacral region on the left side measuring 6.5 x 3.7 x 5.6 cm along with intensely enhancing solid component and multiloculated cystic areas. In addition, a bifid spinous process of L4-L5, S1 was noted. The clinical possibilities considered included lipoma, lymphangioma, meningocele, rhabdomyosarcoma, or sacrococcygeal teratoma.

**Figure 1:**
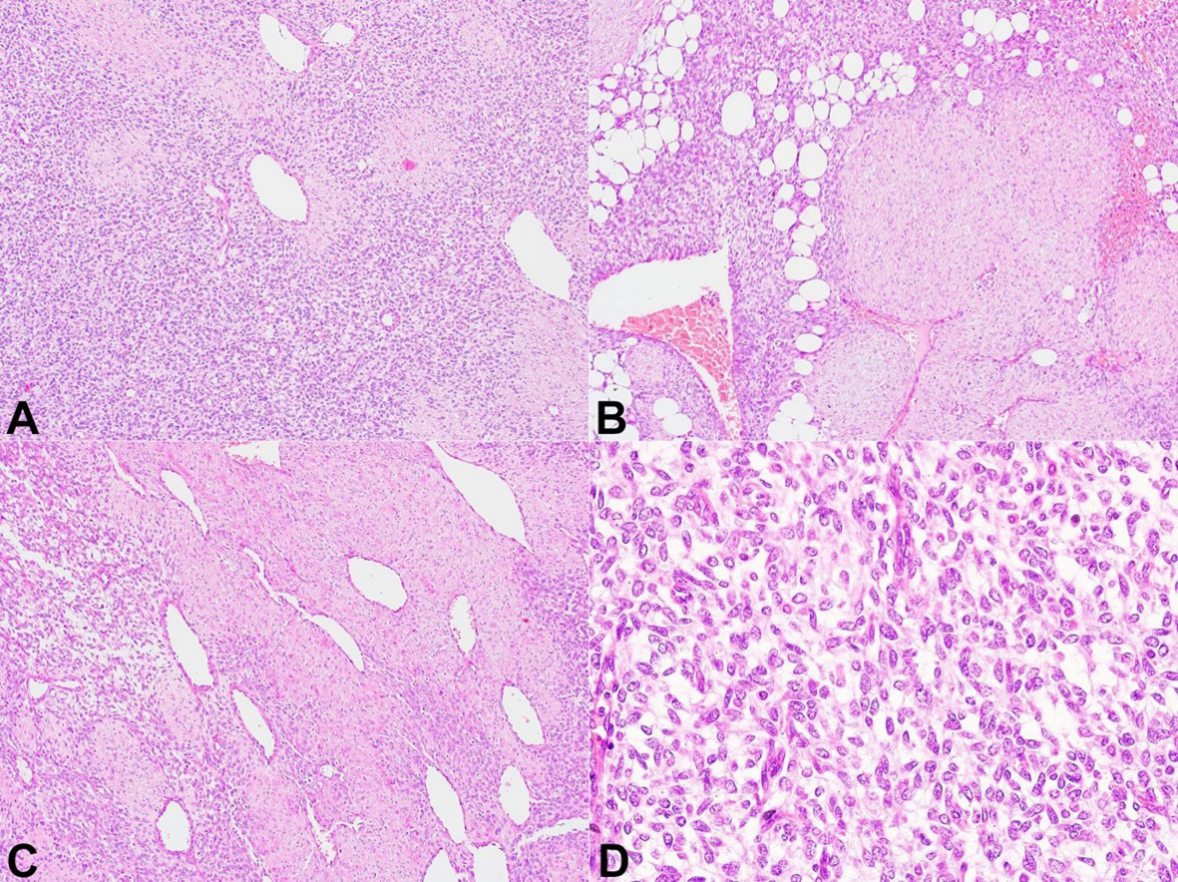
Photomicrographs of the tumor. **A** - Diffuse sheets of immature mesenchyme with intervening blood vessels (H&E x400); **B** - Fascicles of fibroblasts observed in between immature mesenchyme with lobules of adipocytes (H&E x400); **C** - Dilated blood vessels at the periphery of the lesion (H&E x400); **D** - Neoplastic cells constituting the immature mesenchyme depicting round to elongated cells with conspicuous nucleoli and scant cytoplasm (H&E x1000).

The lesion was excised. No capsule could be identified peroperatively. On gross examination, the lesion contained both solid and cystic areas and measured 9x5x3 cm. On microscopy, a poorly circumscribed tumor was identified, which revealed immature mesenchymal cells arranged in long fascicles and trabeculae intercepted by fibrocollagenous tissue composed of bland fibroblasts and myofibroblasts. The tumor also showed lobules of mature adipocytic tissue (Figures 1A and 1B). Few dilated blood vessels, some with stag-horn morphology were observed, more at the periphery of the lesion (Figure 1C). The immature mesenchyme was characterized by round to elongated cells with vesicular chromatin, inconspicuous nucleoli and moderate amount of eosinophilic cytoplasm (Figure 1D).

**Figure 2:**
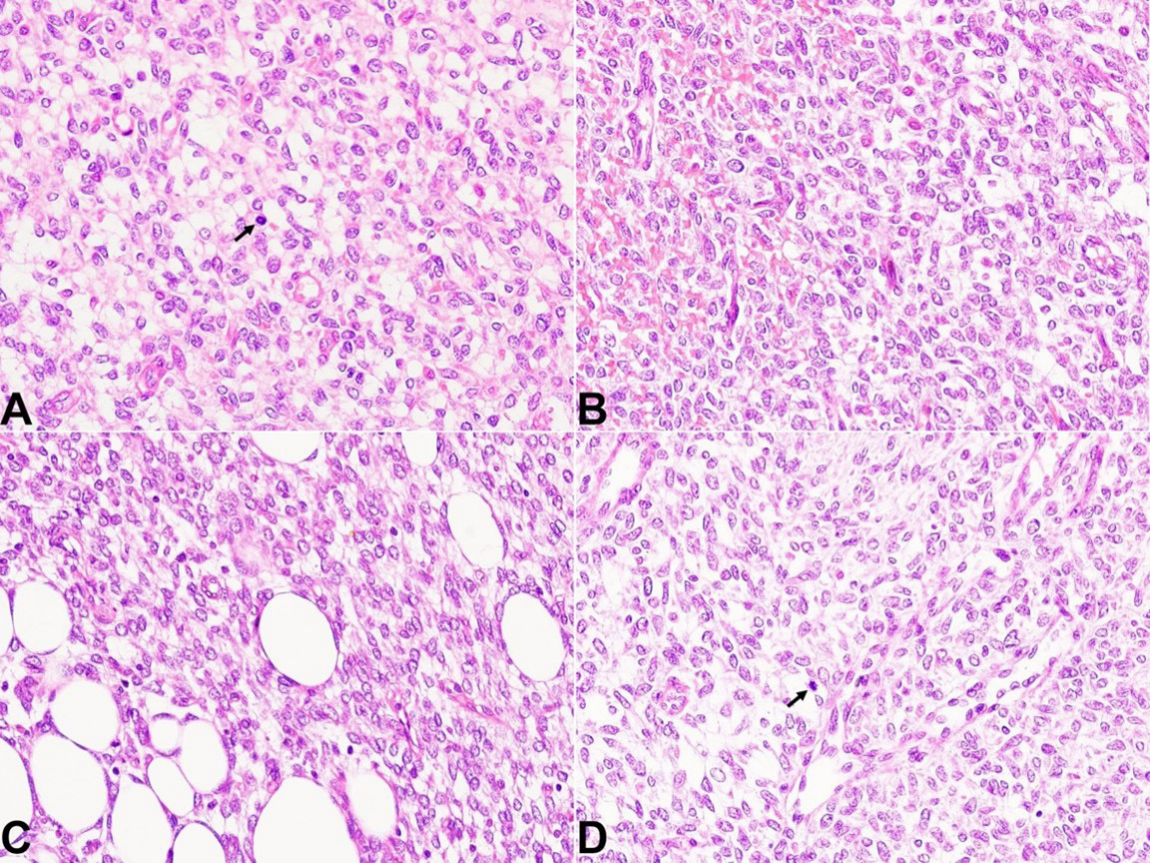
Photomicrographs of the tumor. **A-D** - Foci with sarcomatous morphology. Islands with increased cellularity, nuclear atypia and brisk mitotic activity (arrows) (H&E x1000).

Additionally, there was evidence of sarcomatous transformation with the presence of islands featuring increased cellularity and brisk mitotic activity ranging from (3-4/hpf) (Figures 2A-D).

**Figure 3:**
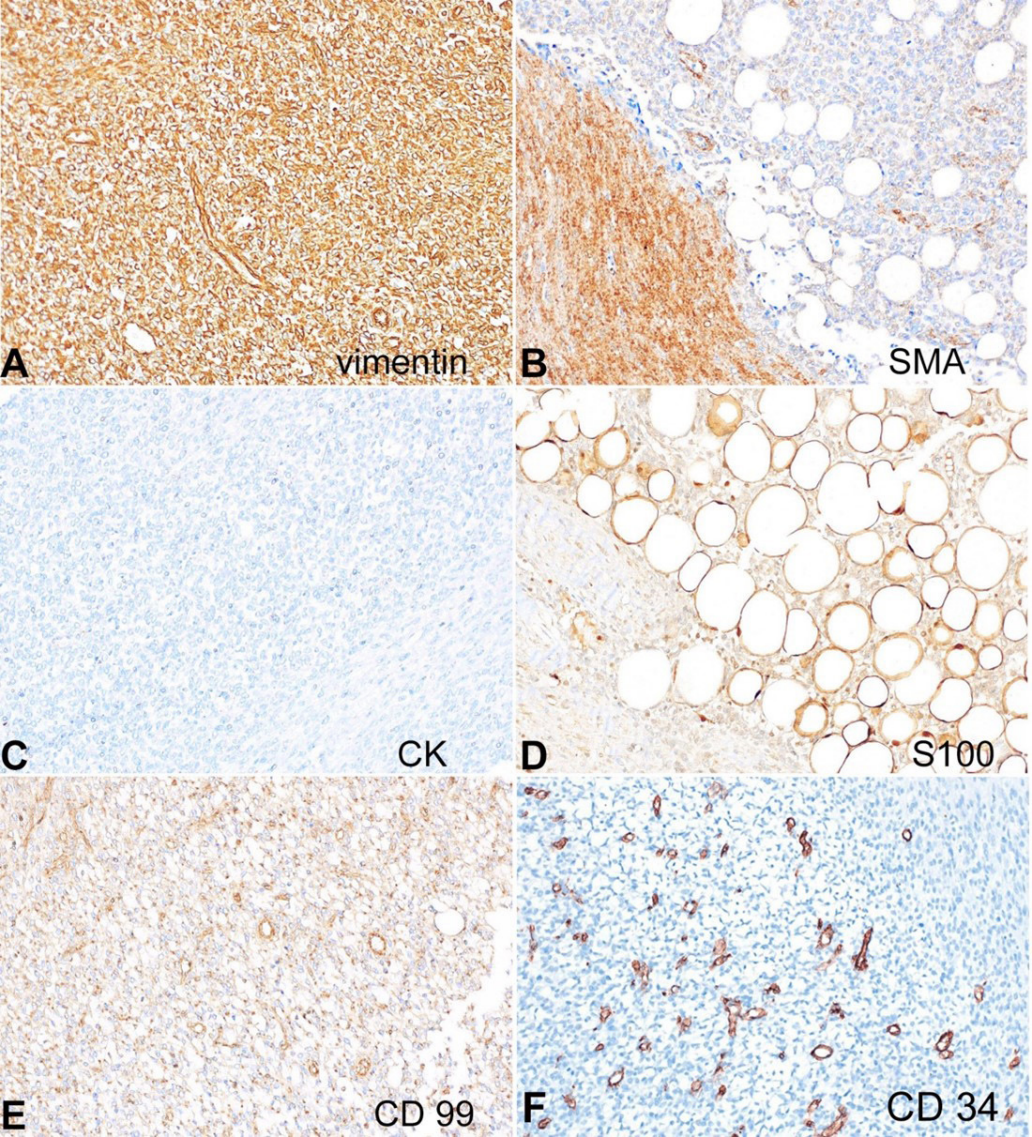
Photomicrographs of the tumor. **A** - Neoplastic cells strongly positive for vimentin; **B** - Smooth muscle actin highlighting the fibroblasts and myofibroblasts; **C** - Neoplastic cells negative for cytokeratin (CK), Melan A, HMB45, Myogenin, Desmin; **D** - S-100 immunostain highlights the adipocytes; **E** - Focal dot-like immunoreactivity observed for CD99; **F** - CD34 highlights blood vessels while the neoplastic cells are negative. (A-F: immunoperoxidase x400).

The immature mesenchyme demonstrated immunopositivity for vimentin. Smooth muscle actin (SMA) highlighted the fibroblasts and myofibroblasts amidst the neoplastic component. CD99 showed diffuse strong perinuclear dot-like positivity. Cytokeratin, myogenin, melan A, desmin, and HMB45 were negative. S-100 highlighted the adipocytes. CD34 highlighted the blood vessels while was negative in all other three components (Figures 3A-F). The tumor was also evaluated for *ETV6-NTRK3* fusion and was found to be negative. Such triphasic morphology favored FHI, albeit with sarcomatous transformation.

Subsequently, a metastatic workup of the child was done, which included a bone scan and CT chest, both of which were normal. The child was started on intermediate-risk rhabdomyosarcoma (RMS) protocol (consisting of vincristine, adriamycin, and cyclophosphamide) in view of the sarcomatous component. Presently, the child has completed 8 weeks of chemotherapy and is disease-free at the last follow-up (two years from the diagnosis).

## DISCUSSION

A rare and unique tumor in infants, FHI commonly arises in axillary regions, upper arms, upper trunk, inguinal region, and external genital areas. Nearly 25% may occur congenitally.^7^ as was seen in the index case. They are commonly confined within the subcutaneous plane and range from 0.5-4cm in diameter. The clinical and radiologic features of this entity are nonspecific, and hence can be confused with malignant soft tissue tumors.^8^^,^^9^ The histogenesis is still unclear with the lesion being referred to as malformation to hamartomatous to a neoplastic one. On histology, it typically shows a triphasic morphology with a variable admixture of primitive mesenchyme, fascicles of fibroblasts/myofibroblasts and adipose tissue. While histology alone is sufficient for diagnosis, immunohistochemistry is a useful adjunct. Electron microscopic and immunohistochemical studies have demonstrated that the two mature components within the lesion comprise fibrous and adipose tissue, while the immature mesenchymal component has primitive fibroblastic features, and lacks muscle or neural differentiation.^10^ In the index case, the mesenchymal component contained sarcomatous-appearing foci with high cellularity, high nuclear grade, and brisk mitotic activity. The occurrence of such high-grade areas is rare and documented in only two cases in a large multi-institutional cases series comprising 145 cases of FHI.^6^ Amongst the two reported cases in their series, one arose in a 6-year-old boy while the other was a congenital lesion. The clinical significance of such morphology remains to be determined as the follow-up was lacking in one of their cases, while the patient with a congenital tumor was treated with radical resection and was reported to be well four years following surgery. A few uncommon cases with large size, rapid growth, infiltrative growth, and/or locally recurrent have also been reported.^11^^,^^12^ Nevertheless, identifying sarcomatous-appearing foci is essential as local recurrence happens in such masses.^6^

The common diagnostic differentials depend on age and the anatomic site. Fibromatosis, lipofibromatosis, rhabdomyosarcoma, teratoma, and congenital fibrosarcoma are differentials for a tumor arising at this location.^13^ Fibromatosis in infants and children occur exclusively in fingers and toes and microscopically, reveals fascicles of spindle cells set against a variably collagenized stroma with infiltrating borders. Lipofibromatosis is characterized by abundant adipose tissue traversed by bundles of fibroblast-like cells and is devoid of any immature mesenchymal component. Rhabdomyosarcoma, the classic ‘small-blue, round cell’ tumor, features sheets of primitive-looking, small round cells with high N/C ratio, which are positive for desmin, myoD1, and myogenic, which were negative in the present case. Teratomas are known to have a variable morphology depending upon the component of the germ layer constituting its bulk. Although rare at this location, congenital fibrosarcoma, a malignant spindle cell tumor, also needs exclusion. They are composed of small, spindle-shaped cells separated by variable amounts of pale stroma, often myxoid in appearance. *ETV6-NTRK3* gene fusion reliably differentiates this tumor from other childhood spindle-cell tumors.^14^

In conclusion, the report illustrates the histomorphology of FHI, which rarely may demonstrate a sarcomatous morphology. Hence, a careful assessment of the lesion is required, and a close follow-up is warranted as local recurrences have been reported.
